# Editorial: Induction of immune tolerance: addressing unmet medical need in immune mediated diseases and immune responses to biologics

**DOI:** 10.3389/fimmu.2023.1219854

**Published:** 2023-08-22

**Authors:** Amy S. Rosenberg, Sophie Tourdot, David Markusic

**Affiliations:** ^1^ EpiVax, Inc, Providence, RI, United States; ^2^ BioMedicine Design, Pfizer, Inc, New York, NY, United States; ^3^ Department of Pediatrics, Herman B Wells Center for Pediatric Research, Gene and Cell Therapy Program, Indiana University School of Medicine, Indianapolis, IN, United States

**Keywords:** immunotherapy, tolerance induction, immunogenicity, anti-drug antibodies (ADA), autoimmune diseases

This Research Topic focuses on 1) strategies for immune tolerance induction in immune mediated diseases and 2) prevention and mitigation strategies for immunogenicity of biological therapeutics.

## Immune tolerance approaches in immune mediated diseases

**Figure 1:**
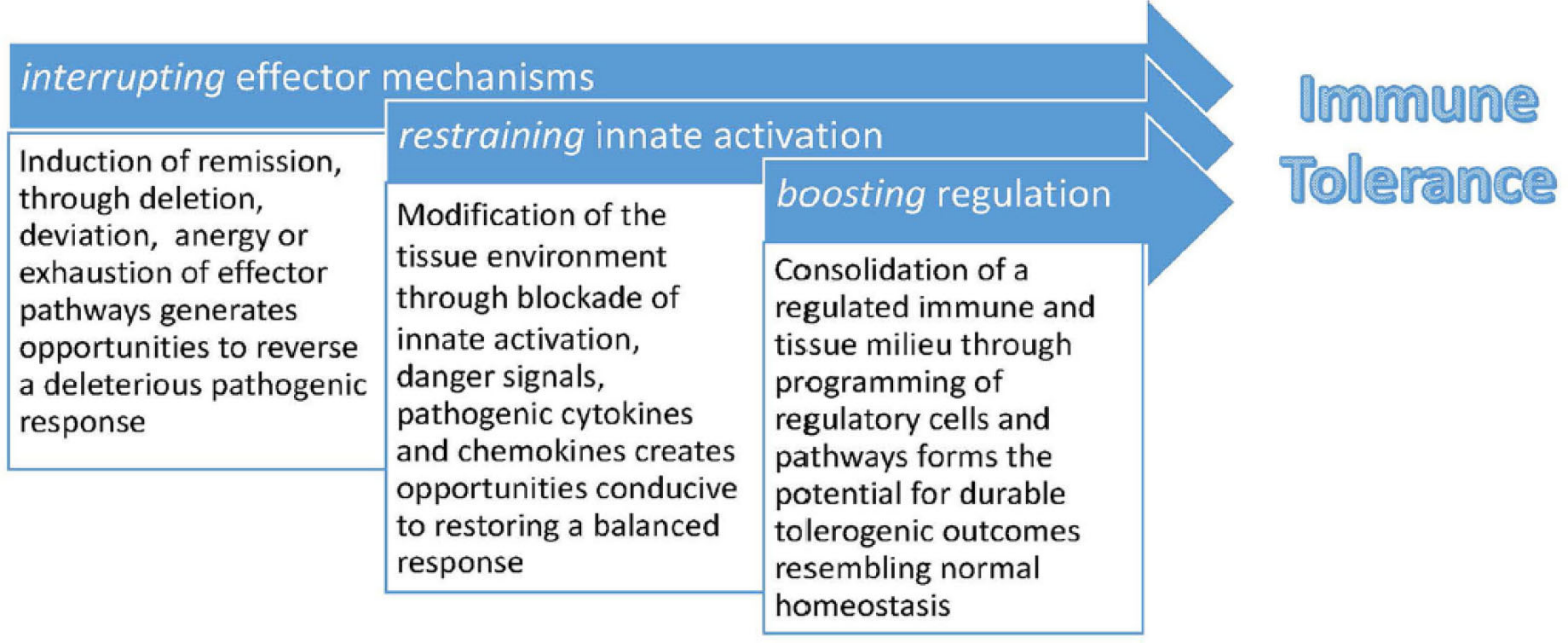
Strategy for immune tolerance induction as elucidated by the Immune Tolerance Network.

The critical overarching strategy for immune tolerance induction in the settings of autoimmunity, transplantation and allergy includes 1) interrupting effector mechanisms 2) restraining innate activation and 3) boosting regulation (**Figure 1**). Moreover, emphasis is placed on safety considerations, the role of epitope spread, and the need for mechanistic studies for informing future studies (Huffaker et al.).

A key concern for inducing antigen specific tolerance using a single agent in the context of pre-existing autoimmunity, allergy, or anti-drug antibodies (ADA) is the possibility of boosting extant responses and worsening disease, realized in a study employing an altered peptide ligand in MS (1). This outcome stressed the importance of developing combinatorial approaches to minimize this possibility. This is illustrated in the multifactorial treatment approaches for allergy and autoimmunity. In allergy, antigen specific desensitization treatments target both IL-4 signaling with Dupilumab, an anti-IL-4R mAb (interrupt effector mechanisms), combined with sublingual grass desensitization (boost regulation); and in the CATNIP trial in which Tezepelumab, an anti-TSLP mAb (interrupt effector mechanisms) is co-administered with Cat Allergen Extract (boost regulation). In Type 1 diabetes (T1D) initial focus was on interrupting effector mechanisms by depleting or modulating Teff cells with Teplizumab (a CD3mAb) (2), or Alefacept (LFA3-Ig) (3). While both induced marked CD8+ T cell exhaustion and prolonged the development of clinical T1D from precursor stages, neither agent changed the level of Tregs, and the patients ultimately developed T1D, further illustrating the need to “boost regulation” in this context. For tolerance to organ transplantation, the need to more effectively delete donor reactive T cells and boost Tregs or Tr1 cells to the relevant alloantigens is paramount as studies promoting tolerance *via* autologous hematopoietic stem cell transplant fail in the majority of patients. Approaches anticipated or in ongoing studies include administration of “tolerance promoting” cell therapies (the ITN TEACH trial), or infusion of alloantigen specific Tregs (ITN LITMUS Trial), and especially promising, engineered Tregs that either express target alloantigens or CARs or TCRs reactive to alloantigens (4).

In this Research Topic, Docampo et al. highlight the need for multimodal approaches in the treatment of relapsing-remitting multiple sclerosis (RRMS). GWAS studies indicate that the vast majority of SNPs associated with MS risk pertain to immune function related genes, especially HLA haplotype (HLA-DR15). Environmental risk factors include Epstein Barr virus (EBV) infection, whose key role has recently been elucidated (5). Brain homing CD4+T cells are the lynchpin in driving proinflammatory B cell and CD8+ cytolytic Teff function, and, activated by MBP, PLP and MOG derived peptides, have a clear functional hierarchy Th1>Th17. Approved therapeutics for RRMS mainly focus on disrupting effector mechanisms and Docampo et al. argues for incorporation of tolerance boosting regimens into existing treatment protocols as evaluated in studies of EAE.

The key principles elucidated by the ITN including safety, the role of epitope spread in disease progression (and tolerance), and the importance of mechanistic studies to define the critical parameters mediating or highly correlating with tolerance induction are discussed by Schurgers and Wraith. Antigen Processing Independent T cell Epitopes (Apitopes) are CD4+ T-cell epitopes that bind directly to MHC II in the conformation recognized by cognate T cells. Moreover, Apitopes bind selectively to “steady state” tolerogenic dendritic cells, the basis for which requires further elucidation.

Tolerance in T1D is explored in several novel approaches in this Research Topic. Sun et al. examined the potential for the regulatory adjuvant, kynurenin, produced by indoleamine 2, 3 dioxygenase (IDO), to increase Tregs in the setting of an autoantigen GAD65 vaccine. With previous studies of GAD65-Alum (Diamyd^®^) reportedly indicating safety, the potential to increase the efficacy of the immunomodulatory vaccine by the addition of kynurenin was investigated. Meanwhile, Zhang et al. modified peptide residues in contact with the TCR and found that substitution of a single amino acid by its D isoform at a TCR contact residue reduced autoreactive CD8 T cell function, T cell organ infiltration, and inflammatory responses in the pancreas. Maulloo et al. investigated induction of antigen specific tolerance through liver targeting of Antigen-N-acetylglucosamine glycopolymer conjugates showing that, as with IV delivery, Treg generation induced robust tolerance when the glycopolymer was administered SC. And finally, Al-Mrahleh et al. used IFNg and TNFa treated Wharton’s Jelly- Derived Mesenchymal Stromal Cells to induce tolerogenic dendritic cells and Tregs in PBMC from T1D patients, upregulating IL-10 and TGF-b, and downregulating IL-17 and IFN-g, but not IL-6. A major limitation of these studies was that they were limited to *in vitro* assays and require further validation under *in vivo* inflammatory conditions.

## Focus on tolerance to therapeutic proteins

In “Driving CARs to BARs: The winding road to specific Regulatory T cells for Tolerance,” Scott provides an outstanding historical review of the development of Treg therapies as well as a critique of individual approaches: TCR vs CAR T regs vs B cell Antigen Receptor (BAR) Tregs (6). BAR Tregs elude issues regarding the HLA-matching restrictions for TCR Treg donor-recipient combinations and conformational epitope matching of CAR T scFV variable domains by expressing on Treg the target antigen to which antigen specific B cells bind, inducing B cell anergy or elimination. Thus, BAR Tregs are appropriate for antibody-mediated disease.

In studies by Lagassé et al. endogenous NK cells were found critical to FVIII tolerance mediated by a FVIII Fc-fusion protein (rFVIIIFc), through its binding to FVIII-specific memory B cells *via* the FVIII moiety and to FcγRIIIA/CD16 on NK cells, which killed FVIII-specific memory B cells *via* ADCC.

ADA to highly effective TNF mAbs in treatment of inflammatory bowel disease (Crohn’s and Ulcerative Colitis) abrogate efficacy in up to 65% of patients, causing devastating adverse events including hospitalization and surgery. As Shakhnovich et al (7) make clear, aside from medication adherence, Therapeutic Drug Monitoring (TDM) is the single, most critical step for both preventing and overcoming immunogenicity in clinical practice. However, it remains to be determined whether immune tolerance to TNF mAbs is induced as well as the underlying mechanisms. Of important note, the authors allege that TDM also has the potential to “reverse immunogenicity” with TDM-based dose adjustments.

The remaining manuscripts in this Research Topic focus on a broad variety of topics related to the monitoring, risk assessment, and treatment of unwanted immune responses to different therapeutic modalities.

De Groot et al. describe a personalized immunogenicity risk assessment (PIMA) tool based on the patients’ mutation and HLA haplotype in the context of Pompe Disease treated with Enzyme Replacement Therapy (ERT). PIMA tools have the potential to better identify patients at risk for ADA formation for other lysosomal storage diseases treated with ERT, thus identifying those who would benefit from prophylactic immune tolerance induction.

Vultaggio et al. discuss approaches to predict, identify the mechanism, and prevent or mitigate ADA mediated hypersensitivity reactions (HSR) directed against biologics, including a broad range of protein-based therapies.

Gross et al. delved into major immunogenicity issues pertaining to Adeno-associated viruses (AAV)-based gene therapies including antibodies to AAV capsid determinants, as well as severe adverse events stemming from complement activation. High priority approaches to address immunogenicity include modification of AAV capsid antigens, drug regimens to limit development of ADA, and strategies to transiently eliminate anti-capsid antibodies.

Balcerek et al. with UCSF investigators reported that release criteria for Treg batches in clinical studies of autoinflammatory disease and organ transplant were met in 88% of 7 phase I trials, indicating a consistent manufacturing process produces cellular products meeting standards for critical product quality attributes. The main factor correlating with level of ex-vivo expansion of Tregs was the starting number of Tregs.

Nabhan, M et al. focused on the mechanisms that underlie the interaction of mAb aggregates with antigen presenting cells, emphasizing that antibody aggregates act as danger signals recognized by innate immune cells, to trigger innate and adaptive immune responses (8).

Shah et al. focused on microneedle delivery of therapeutics or vaccines to facilitate optimal immune enhancing or immunomodulatory strategies, as well as eliminating obstacles to current storage and delivery methods.

Sakowska et al. provided a review of the immune pathways involved in cancer immune evasion and, reciprocally, development of autoimmune disease in which similar molecular players work in opposite directions.

## Author contributions

All authors listed have made a substantial, direct, and intellectual contribution to the work and approved it for publication.
